# Chicken *GHR* natural antisense transcript regulates *GHR* mRNA in LMH cells

**DOI:** 10.18632/oncotarget.12437

**Published:** 2016-10-04

**Authors:** Li Zhang, Shudai Lin, Lilong An, Jinge Ma, Fengfang Qiu, Rumin Jia, Qinghua Nie, Dexiang Zhang, Qingbin Luo, Ting Li, Zhang Wang, Xiquan Zhang

**Affiliations:** ^1^ Guangdong Provincial Key Laboratory of Agro-Animal Genomics and Molecular Breeding, Key Laboratory of Chicken Genetics, Breeding and Reproduction, Ministry of Agriculture, College of Animal Science of South China Agricultural University, Guangzhou 510642, P.R. China; ^2^ Agricultural College, Guangdong Ocean University, Zhanjiang 524088, P.R. China

**Keywords:** chicken, GHR natural antisense transcript, GHR mRNA, liver, LMH cell

## Abstract

Growth hormone receptor (GHR) played key roles in human and animal growth. Both human laron type dwarfism and sex linked dwarf chicken were caused by the mutation of *GHR* gene. In this study, we identified an endogenously expressed long non-coding natural antisense transcript, *GHR-AS*, which overlapped with the *GHR* mRNA (*GHR-S*) in a tail to tail manner. Spatial and temporal expression analyses indicated that *GHR-AS* were highly expressed in chicken liver and displayed ascending with the development of chicken from E10 to 3 w of age. Interfering *GHR-AS* caused *GHR-S* decreasing, accompanied with increasing of the inactive gene indicator, H3K9me2, in the *GHR-S* promoter regions in LMH cells. RNase A experiment exhibited that *GHR-AS* and *GHR-S* can form double strand RNAs at the last exon of *GHR* gene *in vivo* and *in vitro*, which hinted they could act on each other via the region. In addition, the levels of *GHR-S* and *GHR-AS* can be affected by DNA methylation. Compared the normal chicken with the dwarfs, the negative correlation trends were showed between the *GHR-S* promoter methylation status and the *GHR-AS* levels. This is the first report of that *GHR* gene possessed natural antisense transcript and the results presented here further highlight the fine and complicated regulating mechanism of *GHR* gene in chicken development.

## INTRODUCTION

The natural occurred antisense transcripts (Nats), widely transcribed in human and animals, usually interacted with the sense RNA via their complemented regions [1, 2] and involved in various biological processes [3, 4], development and diseases [5], suggesting critical roles of antisense transcripts in mammalian gene expression. In eukaryotes, the double transcribed gene was first found in mouse in 1986 [6], subsequently, they were also identified in avian [7]. Currently, Nats have been found existed in diverse organisms, such as mouse [2], chicken [8], pig [9], sheep [10] and human [1]. A lot of Nats have been reported playing important roles in human diseases and mammalian development, such as *Tsix* [4], *DHRS4-AS* [3] and *AS-IL1α* [11]. However, until now only four gene's Nats have been studied in detail in avian and they were chicken *bFGF* [7]*, IGF-II* [12]*, Collagen a1 (I)* [13] and the programmed cell death 2 [14].

Growth hormone receptor played key roles in human and animal growth. Both human laron type dwarfism [15] and sex linked dwarf chicken [16] were caused by the mutation of *GHR* gene. Chicken *GHR* gene, located in chromosome Z, comprised 8 exons and was highly expressed in liver. Growth hormone (GH), combined with GHR in liver and formed GH-GHR-IGFs signal pathway, affected animal development [17]. Previous studies revealed that chicken *GHR* gene possessed several kinds of sense transcripts by alternatively using different 5′UTR and other ways [17, 18].

In the present study, we identified and characterized a tail to tail Nat of chicken *GHR* gene, *GHR-AS* (*GHR* antisense transcript), in chicken liver. *GHR-AS* is a 4,337 bp spliced and polyadenylated RNA transcribed from 3′UTR of the *GHR* gene in the antisense direction that affects the local epigenetic status of *GHR* gene promoter. *GHR-AS* could protect *GHR* mRNA (*GHR* sense transcript, *GHR-S*) by forming double strand RNAs via exon 8 of *GHR* gene. The results will be beneficial in revealing complex regulation network of *GHR* gene.

## RESULTS

### Identification of a naturally occurring antisense transcript overlapping chicken *GHR* gene

**Figure 1:**
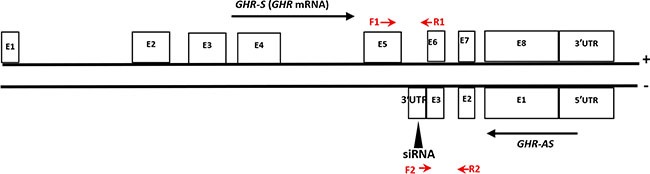
The relative positions of chicken *GHR-AS* and *GHR-S* transcripts in DNA The exons (E) of *GHR-S* (*GHR* mRNA) were noted according to the *GHR* sequence characteristic deposited in Genbank (accession nos.NC_006127.4). Position of the siRNA oligonucleotides was indicated by black triangle. The qPCR primers of *GHR-S* (F1 and R1) and *GHR-AS* (F2 and R2) were labeled with red arrows.

Antisense transcripts were found extensively expressed in chicken liver in our previous study by digital gene expression sequencing of RNA [8]. In addition to the known expressed antisense transcripts, a lot of novel Nats were also detected in our analysis, including *GHR-AS*. Based on the obtained tags of RNA sequencing, by performing 5′ and 3′ RACE, we cloned a 4,337 bp antisense transcript of *GHR*, designated *GHR-AS*, consisting of three exons with a 3′ polyadenylation tail. 5′UTR, exon 1, 2 and 3 of *GHR-AS* overlaps with the 3′UTR, exon 8, exon 7and exon 6 of *GHR* in an antisense type, respectively (Figure 1). The sequence of *GHR-AS* from chicken liver has been submitted to GenBank (accession nos.KX268230). Based on the analysis results of the Coding Potential Calculator (http://cpc.cbi.pku.edu.cn/) and ORF-Finder (http://www.ncbi.nlm.nih.gov/gorf/gorf.html), *GHR-AS* was classified as “noncoding” RNA with a coding potential score of −1.0185 (Supplementary Figure S1).

To verify that *GHR-AS* was an antisense RNA transcribed from the opposite strand of *GHR* gene in chicken liver, by designing biotin-labeled sense or antisense probe, we conducted northern blot. Results indicated that the hybrid signals can be detected at about 4.5 kb position with the sense probe complemented with the *GHR-AS* sequence, and the *GHR* mRNA (*GHR-S*) also can be detected by the antisense probe (Supplementary Figure S2).

### *GHR-AS* and *GHR-S* exhibit similar expression trends within the chicken development

**Figure 2:**
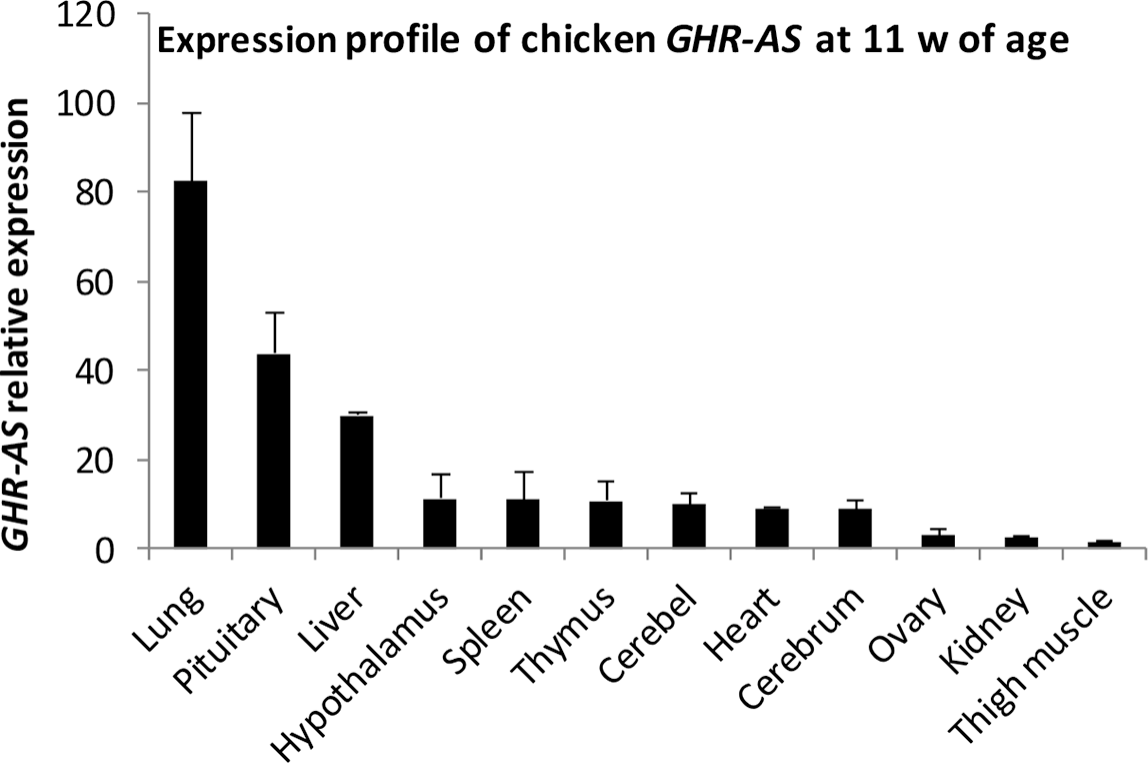
Characterization of the expression profiles of chicken *GHR-AS*

**Figure 3:**
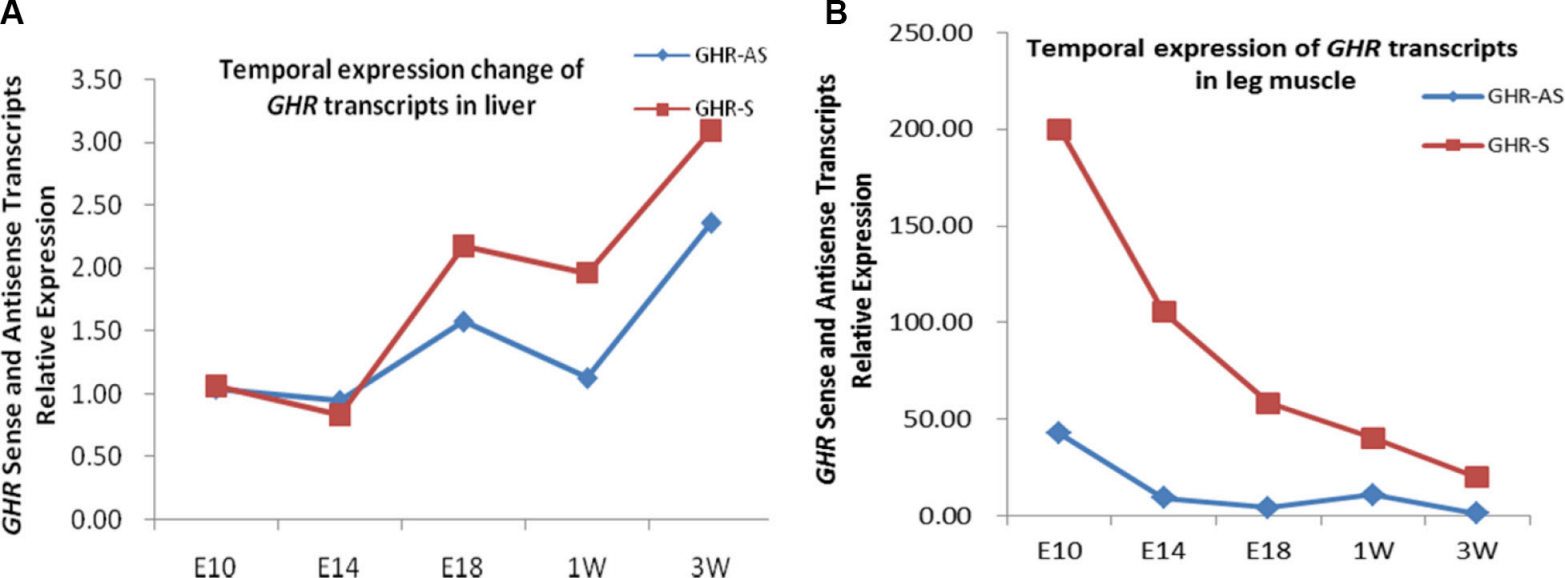
The temporal expression of chicken *GHR-AS* in liver and leg muscle

We examined a panel of RNAs from female chicken tissues at 11 w of age for the presence of *GHR-AS* transcripts by quantitative RT-PCR. Chicken *GHR-AS* transcripts were expressed in all tissues examined, however, there were significant differences in their expression levels. *GHR-AS* were higher in the lung, pituitary and liver than in the other tissues, and it was the lowest in thigh muscle (Figure 2). *GHR-S* played important roles in liver and can form GH-GHR-IGFs signal pathway to affect animal growth [19]. Subsequently, we mainly analyzed the temporal characteristics of *GHR-AS* and *GHR-S* in chicken livers and leg muscles. The *GHR-AS* was expressed more lowly than the *GHR-S* in the two tissues at the indicated points (Figure 3). And the trends of *GHR-AS* expression displayed similarities to those of *GHR-S* in the two tissues. In liver, both *GHR-AS* and *GHR-S* ascended with the development of chicken from E10 to 3 w of age, however they were descendant in leg muscles (Figure 3).

### *GHR-AS* regulates *GHR-S* in chicken LMH cells

**Figure 4:**
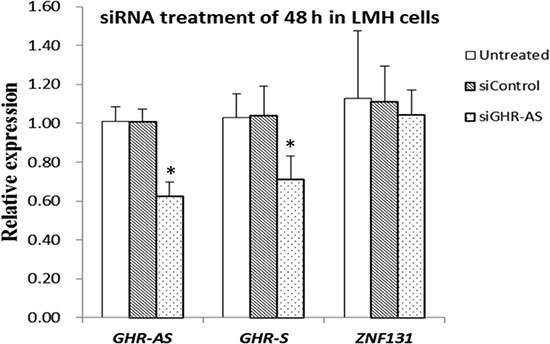
*GHR-AS* regulates *GHR-S* in LMH cells qPCR analysis of the RNA levels of the *GHR-S* after siRNA treatment for 48 h, with the *GAPDH* gene as an internal control. Error bars represent the SEs of three independent experiments.

To check whether the antisense RNA plays roles in *GHR-S* expression, we analyzed the changes of *GHR-S*, after silencing of *GHR-AS* using transcript-specific siRNA that specifically targeted 3′UTR of *GHR-AS* (Figure 1). We firstly measured *GHR-AS* levels in the chicken LMH cells treated with siGHR-AS. Results indicated that siGHR-AS caused visible reductions in *GHR-AS* levels in LMH cell lines. The *GHR-S* RNA expression decreased significantly after the knockdown of *GHR-AS* (Figure 4). To examine whether knockdown of *GHR-AS* affected expression of the nearby *ZNF131* (zinc finger protein 131) gene, we analyzed RNA levels in parallel. Results indicated that knockdown of *GHR-AS* had no effect on *ZNF131* gene expression at the transcriptional level (Figure 4), suggesting that *GHR-AS* specifically regulates the *GHR-S*.

### Silencing *GHR-AS* accompanied with the change of histone modifications in the *GHR-S* promoter

**Figure 5:**
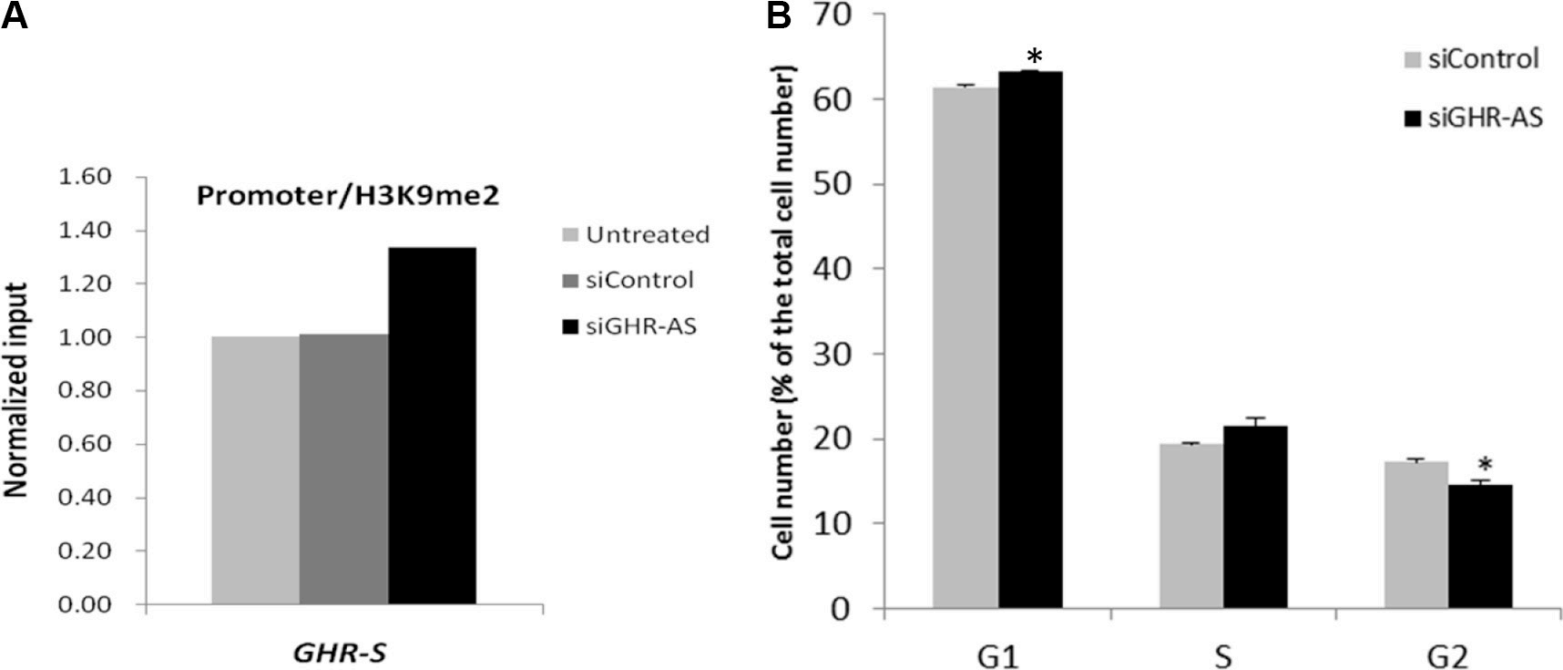
Silencing *GHR-AS* in LMH cell induced an increase of H3K9me2 in the promoter region of *GHR* gene and cell cycle arrest (**A**) ChIP analysis of H3K9me2 enrichment in the *GHR* gene promoters. (**B**) Cell cycle analysis after silencing *GHR-AS*.

To identify a possible mechanism of *GHR-AS* mediated transcriptional inhibition, we analyzed histone modification status in the promoter regions of the *GHR* gene using ChIP assays. We firstly performed western blot to detect the specificity of H3K9me2 antibody and then conducted ChIP assays after knockdown of *GHR-AS* in LMH cells. Inactive gene indicator, H3K9me2, was increased in the *GHR-S* promoter regions in LMH cells (Figure 5A). These results indicated that silencing of *GHR-AS* could change the chromatin status from an open and active state to a closed and inactive state, thereby decreasing *GHR-S* expression. We further examined whether knockdown of *GHR-AS* affected the LMH cell cycles. Results demonstrated that more cells were blocked in G1 phase compared with the control group (Figure 5B).

### *GHR-AS* and *GHR-S* can form an RNA duplex via the last exon of *GHR* gene *in vivo* and *in vitro*

**Figure 6:**
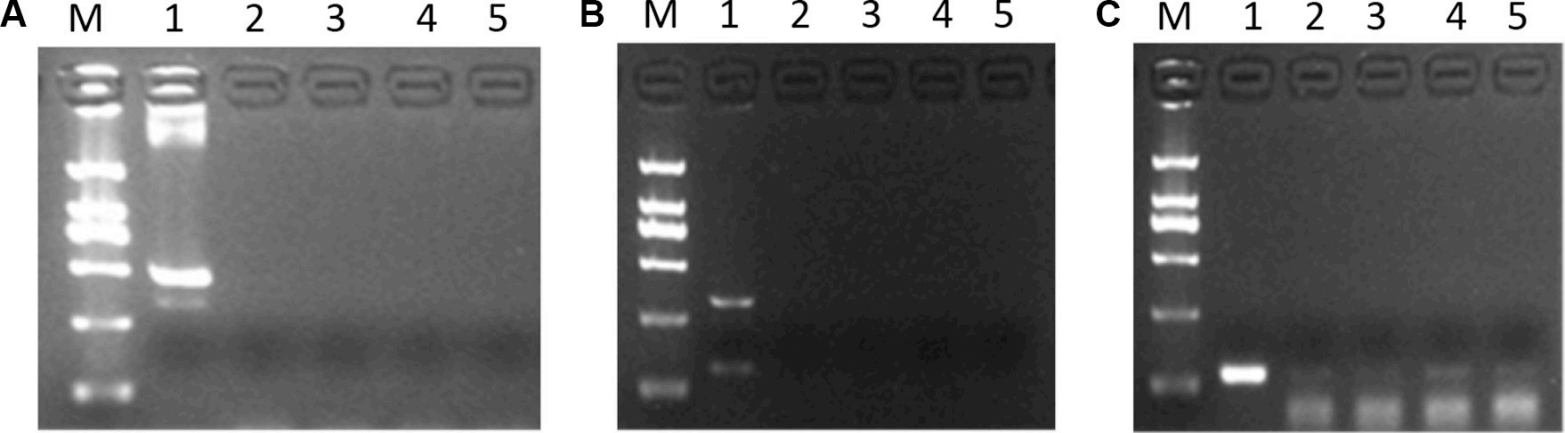
*GHR-AS* and *GHR-S* can form double strand RNAs at the last exon of *GHR* gene (**A**) DNA contamination detection, using β-actin gene, demonstrated the RNAs were DNA-free. 1: positive control using DNA as template for PCR; 2,3: cDNA obtained from RNA of LMH cells; 4,5: cDNA obtained from RNA of chicken liver. (**B**) RT-PCR with gene specific primers, located in exon6 and 7 of *GHR* gene, suggested no production detected. 1: positive control using cDNA as template for PCR; 2,3: cDNA obtained from LMH cells RNA treated with RNase A; 4,5: cDNA obtained from chicken liver RNA treated with RNase A. (**C**) The *GHR-S* and *GHR-AS* RNA hybrids were detected by RT-PCR with gene specific primers, located in exon8 of *GHR* gene. The templates used were the same to (B).

Having manifested the existence of *GHR* sense and antisense transcripts, it was of interest to know whether *GHR-AS* and *GHR-S* can form RNA duplex *in vivo* and *in vitro*. This question was examined by treating total RNA obtained from chicken liver and LMH cells with the single-strand specific nucleases RNase A. The remaining RNA was analyzed by RT-PCR using gene-specific primers which located in different overlapping regions of the both transcripts. Only primers within the exon 8 of *GHR* gene generated a PCR product (Figure 6C) while primers within the exon 6 and 7 of *GHR* gene did not (Figure 6B). Therefore, the overlapping part, exon 8 of *GHR* gene, of both transcripts was protected from degradation. In contrast, *GHR* mRNAs that is not protected by *GHR* antisense RNAs were totally degraded.

### DNA methylation affects both *GHR-AS* and *GHR-S* transcription

**Figure 7:**
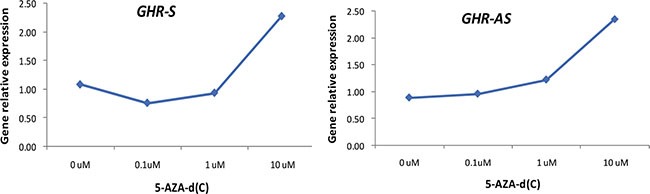
Both *GHR-AS* and *GHR-S* increased with the concentration ascending of 5-AZA-d(C) in LMH cells

**Figure 8:**
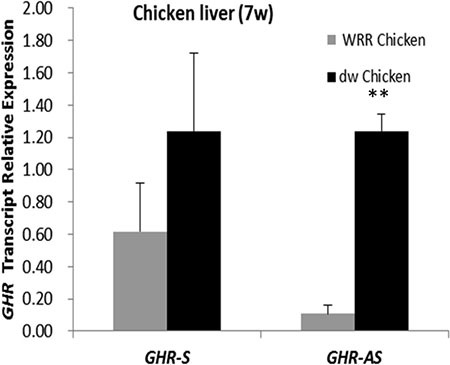
*GHR-S* and *GHR-AS* were highly expressed in dwarf chicken liver than in normal one WRR Chicken: normal chicken; dw Chicken: dwarf chicken

To determine whether the expression of *GHR-AS* and *GHR-S* was related to DNA methylation, we treated LMH cells with 5-AZA-d (C) (Sigma), the inhibitor of DNA methyltransferase, at the concentration of 0 μM, 0.1 μM, 1 μM and 10 μM for 48 h. Quantitative RT-PCR results indicated that both *GHR-AS* and *GHR-S* increased with the concentration ascending of 5-AZA-d(C) (Figure 7). Both *GHR-AS* and *GHR-S* increased at least 2 folds when adding 10 μM of 5-AZA-d(C) in DMEM which indicated that their expression were regulated by DNA methylation. Bioinformatic analysis indicated that CpG islands were rich in the predicted promoter of *GHR-S* but no CpG island was found in the promoter of *GHR-AS* (Supplementary Figure S3). The CpG islands were located in the regions spanning −67,909 to −66,733 bp of *GHR* relative to the A of the initiation codon (accession nos.NC_006127.4), and they were predicted using the Browser (http://www.urogene.org/cgi-bin/methprimer/methprimer.cgi). Further, we conducted DNA methylation analysis on the promoter of *GHR-S* in normal and dwarf WRR female chicken livers at 7 w of age. The CpG sites of the *GHR-S* gene promoter were highly methylated in normal WRR chicken livers, with the lower *GHR-S* and *GHR-AS* expressions while in dwarf WRR chicken, they were lowly methylated, with higher *GHR-S* and *GHR-AS* expressions (Table 1, Figure 8).

**Table 1:** DNA methylation comparison analyses on the promoter of GHR-S between normal and dwarf WRR female chicken livers

Chicken	individual (1)	individual (2)	individual (3)	X ± std	*P*
Normal WRR	56.31%	57.41%	50.52%	54.73% ± 3.71%	0.0075
Dwarf WRR	23.20%	19.81%	22.80%	21.93% ± 1.86%	

The percentage represents the methylated CpG sites out of the total CpG number in the promoter of *GHR-S*.

## DISCUSSION

GHR, expressed highly in the liver, was important in human and animal growth. In this study, we characterized *GHR-AS*, the Nats of chicken *GHR* gene, which were highly expressed in chicken lung, pituitary and liver at 11 w of age. According to the previous report [20], *GHR-AS* overlapped with *GHR-S* in a tail to tail manner. The transcription start site of *GHR-AS* resided in the 3′UTR of *GHR-S* while the AATAA motif and 3′polyadenylation tail of *GHR-AS* were located in the intron 5 of *GHR* gene. Generally, mRNAs were formed by splicing the exons and adding an extra polyadenylation tail. But *GHR-AS* was different from that, and it used the polyadenylation sequences from the intron 5 of *GHR* gene. Coincidentally, an alternative splicing of *GHR* sense transcript used the polyadenylation sequences from the exon of *GHR* gene as its tail to form a 0.7 kb transcript [18]. The *GHR-AS* and the 0.7 kb *GHR-S* transcripts, expressed in liver simultaneously, transcribed from the opposite directions and stopped at the site before they met. It may be related with RNA polymerase II complexes collide, known as “transcriptional interference” [21].

Chicken *GHR* gene was about 80 kb, with 7 polyadenylation sequences located in the DNA region, and possessed multiply transcripts. Both previous studies and this study indicated that chicken *GHR* gene can generate various transcripts via different ways, such as by using the alternative 5′UTR [17], different terminal signals [18] and transcribing from different directions. The function and significance of gene introns were controversial problems. Chicken *GHR-AS* used the intron 5 of *GHR* gene as its 3′UTR to end its transcription, which reminded us that the intron was functional sequences but not the junk DNA and it can be transcribed and used by organism when needed. In this study the intron 5 helped *GHR* gene to generate *GHR-AS* transcript. Hence, this study added a new case for explaining that the intron was functional sequence.

Most sense and antisense RNA pairs showed positive correlations in various tissues [22, 23]. But other studies showed that sense and antisense RNA pairs can be expressed in opposite trends [3, 4, 24, 25]. In chicken liver and leg muscle, *GHR-AS* and *GHR-S* exhibited positive correlations, and espectially in dwarf chicken they were simultanously increased compared with the normal one. The *GHR-AS* was expressed ascendently in liver with the chicken development but was expressed desendently in leg muscle that implied that *GHR-AS* may play different roles in different tissues. It may act activate roles in myoblast fiber formation in ealier chicken embyro development for its higher expression in E10, the important time for the myoblast fiber formation, than in 3 w of age [26].

Until now, most of studies suggested that antisense RNA can regulate sense RNA by the three ways. Firstly, sense and antisense transcripts may potentially interact through their complementary regions, resulting in double-stranded RNA structures that promote stability, transport, and/or translation of the sense transcript [27]. Secondly, antisense RNA can mediate the sense RNA suppressive or active by epigenetic regulation [28]. Thirdly, antisense RNA protects and transports the sense RNA from cell nucleus to the cytoplasm [29]. In this study, interfering *GHR-AS* caused *GHR-S* decreased, accompanied with the inactive gene indicator, H3K9me2, increasing in the *GHR-S* promoter regions in LMH cells. It indicated that *GHR-AS* could regulate *GHR-S* by modifying the histone epigenetic marker and hence affected the cell cycles. It needs to further study whether H3K4me2 can be affected by *GHR-AS* or not.

Several reports showed that the sense/antisense RNA duplex protected the sense RNA via their complementary sequences [30–32]. RNaseA experiment indicated that *GHR-AS* and *GHR-S* can form double strand RNAs at the last exon of *GHR* gene *in vivo* and *in vitro* which hinted they could act on each other through the region. In addition to the exon, the 5′UTR of *GHR-AS* was complemented with the 3′UTR of *GHR-S*. Our group has proved that chicken *GHR-S* 3′UTR was the target of Let-7b [33]. Consequently, the 3′UTR of *GHR-S* may be the competitive binding site for *GHR-AS* and Let-7b if the double strand *GHR-S/AS* RNAs can be formed in 3′UTR of *GHR-S*. Further, many long noncoding RNAs have been confirmed regulating gene mRNA stability via competing for binding to miRNA [34].

DNA methylation is generally considered as repressive epigenetic modification and is critical for the control of gene expression in animals. In this study, both *GHR-S* and *GHR-AS* were risen with the 5-AZA-d(C) concentration increasing, which indicated that the levels of *GHR-S* and *GHR-AS* can be affected by DNA methylation. Antisense transcripts have been proposed to cause DNA methylation. *Tsix* and *AS1DHRS4*, long antisense transcripts, induce DNA methylation at the promoter of the sense transcript *Xist* and *DHRS4L2*, respectively [3, 4]. Moreover, some antisense transcripts have coordinated expression with its counterpart sense gene promoter methylation [22]. In this study, methylation of *GHR-S* promoter was significantly higher in normal chicken liver, coupled with lower *GHR-AS* levels, while the situation was the opposite in dwarf chicken, exhibiting lower DNA methylation status in *GHR-S* promoter and higher *GHR-AS* expression. This implies that *GHR-S* and *GHR-AS* transcripts are not regulated independently but are regulated sufficiently to give simultaneous activation or inactivation of the gene pair. A cis-acting co-regulatory mechanism could exist that is working in a coordinated manner between sense and antisense transcription.

**Figure 9:**
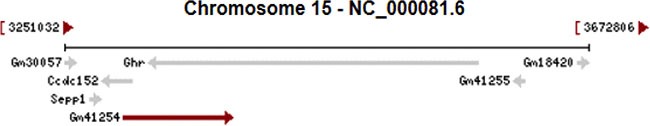
The direction of mouse *GHR* gene and the non-coding transcript Gm41254 The figure was cited from website (http://www.ncbi.nlm.nih.gov/gene) and the arrows represent the directions of transcripts from different genes.

Many genes, such as *BDNF* [5], *XIST* [4] and *DHRS4* [3], were bidirectionally transcribed in various species which implied that the transcribing directions of gene were conserved and the Nats play key roles in gene regulation and biological process. In human and animals, *GHR* genes are highly conserved in structure and function. GH exerts growth-promoting and metabolic effects in target tissues by binding to the transmembrane GHR and triggering enhanced GHR association with, and activation of, the cytoplasmic tyrosine kinase JAK2 [35, 36]. By checking website (http://www.ncbi.nlm.nih.gov/gene), we found the DNA region containing mouse *GHR* gene also exhibited transcribing in both directions. The mouse noncoding transcript Gm41254 showed an opposite direction to *GHR* mRNA (Figure 9). It suggested that *GHR* gene may be transcribed in double directions in various species and *GHR-AS* may act an important role in mammalian and avian growth.

## MATERIALS AND METHODS

### Ethics statement

The Animal Care Committee of South China Agricultural University (Guangzhou, People's Republic of China) approved this study (approval number SCAU#0017). The animals involved in this study were humanely sacrificed as necessary to ameliorate suffering.

### Animals and samples

#### Animals

Recessive White Rock chickens, provided by Guangdong Nanhai Poultry Company (Guangdong, China), were used in this study. Two strains, including normal WRR and dwarf WRR chickens, were used to compare DNA methylation and the expression of the two transcripts. Dwarf chickens had a 1,773 bp deletion mutation at the last exon and in the 3′UTR of *GHR* gene. The weight of dwarf chickens was about 30% less than that of normal chickens. The two strains were fed under the same conditions to 7 w of age.

### RNA extraction and cloning chicken *GHR-AS* by RACE technology

Total RNA was extracted from chicken liver at 7 w of age using the Trizol reagent (Invitrogen, USA) and was tested by agarose electrophoresis. Rapid amplification of the 3′ or 5′cDNA ends (RACE) was conducted using the RACE kit (Clontech, Japan) as described by the manufacturer, followed by nested PCRs of the cDNA copies. The PCR products were cloned and sequenced in the forward and reverse directions. All RACE primers were listed in Table 2.

**Table 2:** Primers used in this study

Application	Primer name	Primer sequence	Tm (°C)
3′RACE	GHR-AS3(GSP1)	GGGTCAATCCCTTTAATCTTT	52
	GHR-AS3(NGSP1)	CAACAACTAAGAACCAGGGAAA	
5′RACE	GHR-AS5(GSP1)	TCCTCCTGTGCCAGTTCC	52
	GHR-AS5(NGSP1)	GCGTGTTCAGGAGCAAAGCT	
GHR-AS full length PCR	GHR-AS-F	TTTTTTTTTTTTTTTTTT	52
	GHR-AS-R	GTATGAAGAGTCCCAACCAAC	
GHR-AS quantitative PCR	qGHR-AS-F	TTGCTAATGTTTCTGTTCTGTG	56.3
	qGHR-AS-R	GGGTCAATCCCTTTAATCTTT	
GHR-S quantitative PCR	qGHR-S-F	AGTCCGATCAAGACAACGTAC'	56.3
	qGHR-S-R	CTAAGAACCAGGGAAACTCG	
ZNF131 quantitative PCR	ZNF131-F	ATGTCCAAACTGCCACG	56.3
	ZNF131-R	CACGCTGTTACAAACCTGA	
DNA contamination detection	β-actin-F	TCATTGTGCTAGGTGCCA	50–60
	β-actin-R	CCTCTTCCAGCCATCTTT	
Internal control in qPCR analysis	GAPDH-F	TCCTCCACCTTTGATGCG	50–60
	GAPDH-R	GTGCCTGGCTCACTCCTT	
Overlapping region(1) detection	OL1-F	TTTCCCTGGTTCTTAGTTGTTG	55
	OL1-R	GGGTCAATCCCTTTAATCTTT	
Overlapping region(2) detection	OL2-F	GCGTGTTCAGGAGCAAAGCT	60
	OL2-R	TGGGACAGGCATTTCCATACTT	
ChIP-qPCR	GHR-S promoter-V1-F	GCCTATAGCTGTCGCCTA	60
	GHR-Spromoter-V1-R	GGAGAGCACTGTCTGATG	
DNA methylation analysis	GHR- S promoter-BSP-F1	GAGGGCGGTCGTTGTTCG	60
	GHR- S promoter-BSP-R1	TTCTCCGCAACGCCCGCTCGTC	
	GHR- S promoter-BSP-F2	GTGATATTTAAGTAAAATAAATTGT GGGAT	66–56 (−0.5°C /cycle)
	GHR- S promoter-BSP-R2	ACTC**R**AC**R**CATTCCTAAAAAC**R**C**R** ACTAACC	

### Northern blot analysis

Northern blotting was employed to identify the expression of *GHR-AS* RNA in the chicken livers. The sense and antisense RNA probes were designed to minimize non-specific hybridization against mRNAs following homology searches using the Basic Local Alignment Tool (BLAST; http://blast.ncbi.nlm.nih.gov/). The selected probe sequence for the detection of *GHR-AS* and *GHR-S* RNA were listed in Table 3. The sense and antisense probes were synthesized and labeled with biotin (Sangon Biotech Company, China). The total RNA, obtained from chicken livers, was DNA removed and transferred to a nylon membrane and UV-crosslinked. The membranes were pre-hybridized with alkaline-denatured salmon sperm DNA and then probed with biotin-labeled sense or antisense probe. The membranes were washed with wash buffer A for 10 min at 25°C and then washed twice with wash buffer B for 20 min at 60°C (Beyotime Biotechnology, China). Signals were visualized on a phosphorimager machine.

**Table 3:** The single strand probes, biotin labeled, used for Northern blot to detect GHR-Sand GHR-AS

Application	Name	Probe sequence
GHR-S detection	GHR-antisense probe	5′-TCAATCCCTTTAATCTTTGGAACTGGCACAGGAGGAAAAATCA GCATTTTTAACCTTGGCTGTTTAGACAACAGGATTAAGATCGCTGT TACGGCCAGCCCACACACT −3′
GHR-AS detection	GHR-sense probe	5′-GCGTGTTCAGGAGCAAAGCTGTAACGAGGACACTTACTTCACC ACAGAAAGCCTTACCACTACCGGTATCAATCTTGGAGCTTCAATG GCAGAAACCCCAAGTATGG-3′

### Reverse transcription and quantitative PCR

Reverse transcription reaction was performed using PrimeScript RT reagent Kit (Perfect Real Time) (Takara, Otsu, Japan) as described in the manufacturer's protocol. Real time PCR was conducted in anBio-rad CFX96 Real-Time Detection system (Bio-rad, Hercules, CA, USA) using KAPA SYBR FAST qPCR Kit (KAPA Biosystems, Wobrun, MA, USA). All primers were listed in Table 2. Gene expression was normalized with *GAPDH*.

### Cell lines and culture conditions

We cultured chicken LMH cells, gifted from China Harbin Veterinary Research Institute, in high-glucose Dulbecco's modified Eagle's medium (Gibco, Grand Island, NY, USA) with 10% (v/v) fetal bovine serum (Hyclone, Logan, UT, USA) and 0.2% penicillin/streptomycin (Invitrogen, Carlsbad, CA, USA).

### RNA interference

LMH cells were transfected with 50 nM of siRNAs targeting *GHR-AS* using lip3000 kit (Invitrogen, USA) according to the manufacturer's direction. Total RNA was harvested 48 h later for Real-time quantitative PCR analysis. The siRNA sequence was listed in Table 4.

**Table 4:** siRNA targeting chicken GHR-AS

siRNA name	Sequence (5′–3′)	
	Sense	Antisense
siRNA-GHR-AS (siGHR-AS)	GCAUCUCUGUUGCAUCUUUTT	AAAGAUGCAACAGAGAUGCTT
Negative control (siControl)	UUCUCCGAACGUGUCACGUTT	ACGUGACACGUUCGGAGAATT

### Cell cycle distribution analysis

In order to determine the effects of *GHR-AS* on the cell cycle distribution, the cells were collected 48 h after siRNA transfection. The cells were washed twice with ice cold PBS and fixed with 70% ice-cold ethanol overnight at −20°C until further processing. After incubation in 50 *μg*/mL propidium iodide (Sigma Life Science, St. Louis, MO, USA) containing 10 *μg*/mL RNase A (Takara, Otsu, Japan) and 0.2% (v/v) Triton X-100 (Sigma) for 30 min at 4°C, the cells were analyzed using a FACSAriaII flow cytometer (BD Biosciences, San Jose, CA, USA) and ModFit Lt 4.1 software (Verity Software House, Topsham, ME, USA).

### Characterization of double-stranded RNA

Total RNA was treated with DNase I (Takara, Otsu, Japan) in DNase I buffer according to the manufacturer's instructions until no traces of DNA could be detected by PCR analysis. DNA-free total RNA was digested with RNase A (Takara, Otsu, Japan) at a terminal concentration of 20 ng/*μ*L at 37°C for 1 h according to the manufacturer's instructions. Following the RNase A protection assay, we used RT-PCR to detect duplex formation by gene-specific primers located in different overlapping regions (the primers are listed in Table 1).

### Chromatin immunoprecipitation (ChIP) assays for histone 3 lysine 9 methylation

Standard ChIP assays were performed using the Pierce™ AgaroseChIP Kit (Thermo Fisher Scientific, USA) according to the manufacturer's instructions. LMH cells were harvested after transfected with siRNAs for 48 h to reach 90% confluence. The cross-linking between the nuclear protein and genomic DNA was performed in the growth medium with 1% formaldehyde supplementation at room temperature for 10 min. The harvested cells were lysed with Lysis Buffer I. Chromatin was digested with MNase for 15 min at 37°C and incubated with dimethylated H3K9 antibodies (Abcam, Cambridge, UK) or normal rabbit IgG overnight at 4°C. The antibody-chromatin complex beads were captured by Protein A/G agarose. DNA was separated from the beads and eluted using IP Elution Buffer for 40 min at 65°C. The DNA was further purified using a DNA Clean-up Column. Immunoprecipitated DNA was analyzed using real-time PCR, with gene-specific primers for *GHR-S* promoter, normalized by input DNA.

### Bisulfite sequencing for DNA methylation analysis

Genomic DNA of chicken livers was extracted with a DNA tissue kit (Omega, USA), and the bisulfite conversion reaction was performed using an EpiTect Bisulfite kit (Qiagen, Germany) according to the manufacturer's instructions. PCR amplification of bisulfate-treated DNA was respectively carried out with methylated site primers (listed in Table 1). The amplified products were cloned and sequenced. For each PCR product, more than eight clones were sequenced to analyze the *GHR* gene methylation status in the promoter region.

### Statistical analysis

Data were processed using the statistical software package SAS 9.1.3 (SAS Institute Inc., NC) and expressed as the mean ± SE. Variance analysis was completed using a GLM procedure. *P* < 0.05 was considered a significant difference between the groups.

## SUPPLEMENTARY MATERIALS FIGURES


