# Supplementary material for: Chicken GHR natural antisense transcript regulates GHR mRNA in LMH cells
Source: Oncotarget. 2016 Oct 4;7(45):73607–17. doi: 10.18632/oncotarget.12437 (PMC5342002; doi:10.18632/oncotarget.12437)
Supplement: Supplementary file 1 [file oncotarget-07-73607-s001.pdf]

Chicken *GHR* natural antisense transcript regulates *GHR* mRNA in LMH cells

Supplementary Materials

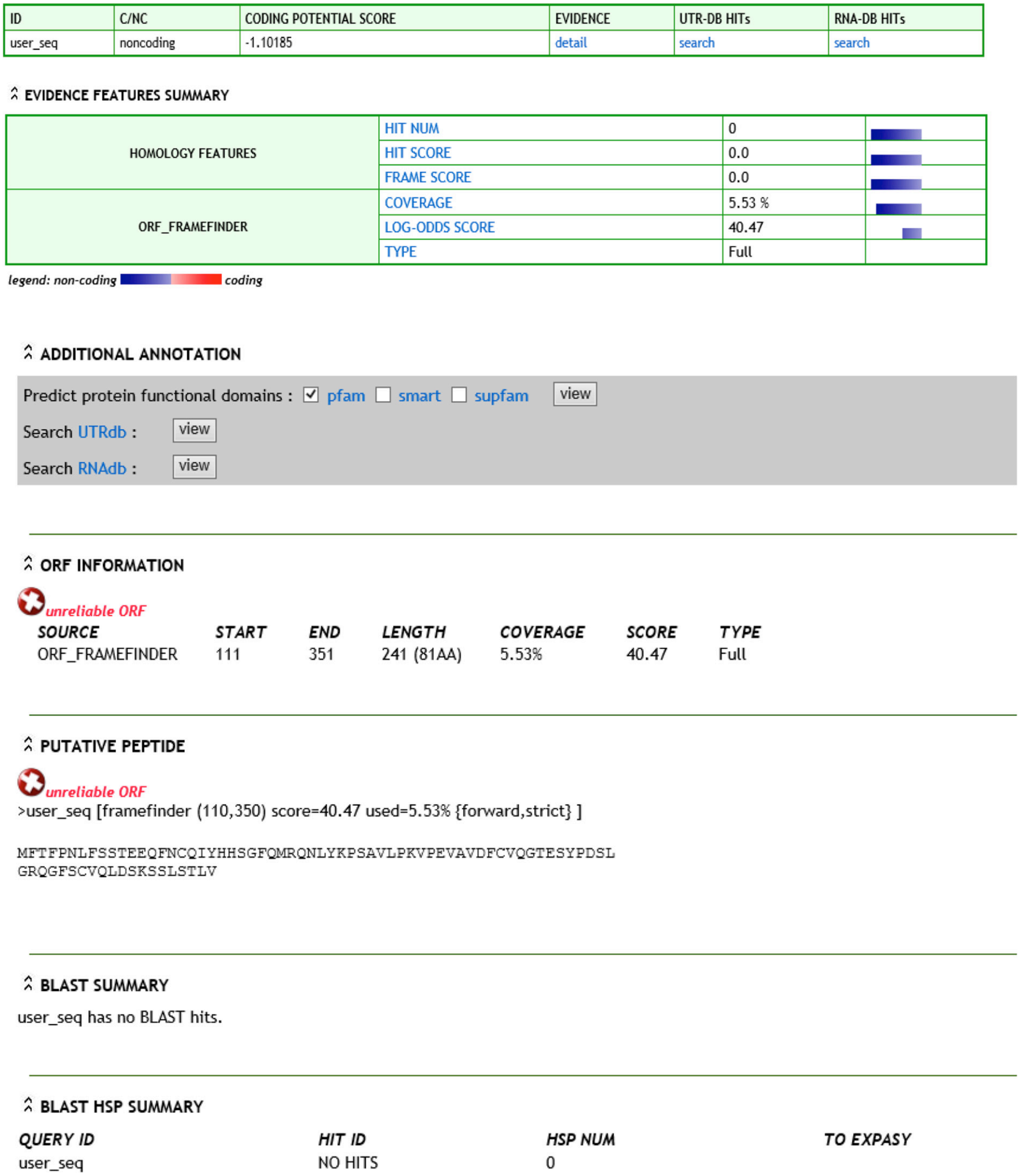

ID	C/NC	CODING POTENTIAL SCORE	EVIDENCE	UTR-DB HITs	RNA-DB HITs
user_seq	noncoding	-1.10185	detail	search	search

EVIDENCE FEATURES SUMMARY

HOMOLOGY FEATURES	HIT NUM	0	
	HIT SCORE	0.0	
	FRAME SCORE	0.0	
ORF_FRAMEFINDER	COVERAGE	5.53 %	
	LOG-ODDS SCORE	40.47	
	TYPE	Full	

legend: non-coding coding

ADDITIONAL ANNOTATION

Predict protein functional domains : pfam smart supfam view

Search UTRdb : view

Search RNAdb : view

ORF INFORMATION

unreliable ORF

SOURCE	START	END	LENGTH	COVERAGE	SCORE	TYPE
ORF_FRAMEFINDER	111	351	241 (81AA)	5.53%	40.47	Full

PUTATIVE PEPTIDE

unreliable ORF

>user_seq [framefinder (110,350) score=40.47 used=5.53% {forward,strict}]

MFTFPNLFSSTEEQFNCQIYHHSGFQMRQNLYKPSAVLPKVPEVAVDFCVQGTESYPDSL
GRQGFSCVQLDSKSSLSTLV

BLAST SUMMARY

user_seq has no BLAST hits.

BLAST HSP SUMMARY

QUERY ID	HIT ID	HSP NUM	TO EXPASY
user_seq	NO HITS	0	

Supplementary Figure S1: Analysis for the protein coding capacity of *GHR-AS*. The *GHR-AS* sequence was analyzed using Coding Potential Calculator (http://cpc.cbi.pku.edu.cn/). Results indicated *GHR-AS* was classified as "noncoding" RNA with a coding potential score of –1.0185.

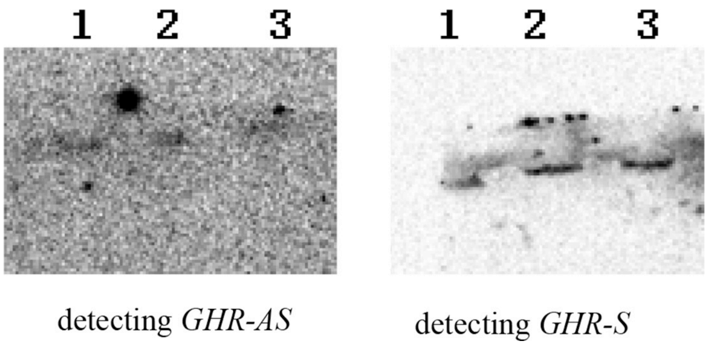

Supplementary Figure S2: Northern blot were conducted to detect *GHR-AS* and *GHR-S* 1, 2 and 3 were total RNA extracted from 3 individual chicken livers at 7 w of age. Northern blotting was employed to identify the expression of *GHR-AS* and GHR-S RNA in the chicken livers. The sense and antisense RNA probes were designed biotin labeled (Sangon biotech company, China). The hybrid signals can be detected at about 4.5 kb position with the sense probe complemented with the *GHR-AS* sequence, and the *GHR* mRNA (*GHR-S*) also can be detected by the antisense probe.

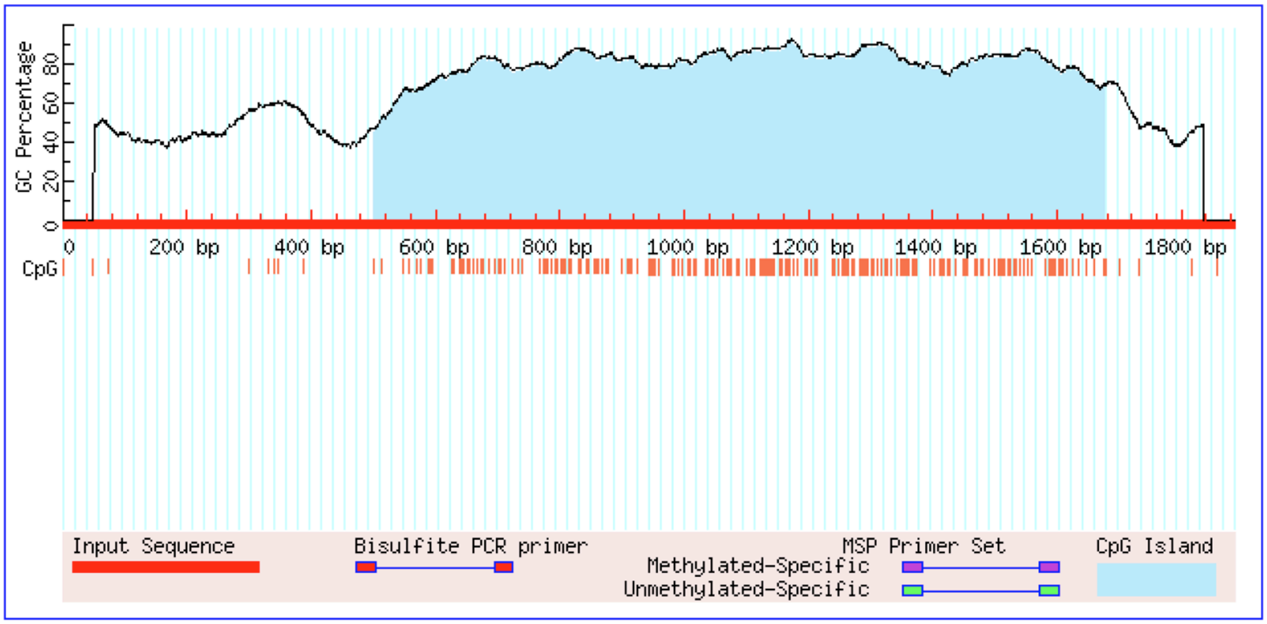


```
Sequence Name:
Sequence Length: 1884

CpG island prediction results
 (Criteria used: Island size > 100, GC Percent > 50.0, Obs/Exp > 0.6)
  1 CpG island(s) were found in your sequence
              Size          (Start - End)
  Island 1    1176 bp        (501 - 1676)
```

Supplementary Figure S3: CPG island analysis in *GHR-S* promoter. Bioinformatic analysis indicated that CpG islands were rich in the promoter of *GHR-S* (http://www.urogene.org/cgi-bin/methprimer/methprimer.cgi). The CpG islands were located in the regions spanning –67,909 to –66,733 bp of *GHR* relative to the A of the initiation codon (accession nos.NC_006127.4).
